# The Effects of Direction of Exertion, Path, and Load Placement in Nursing Cart Pushing and Pulling Tasks: An Electromyographical Study

**DOI:** 10.1371/journal.pone.0140792

**Published:** 2015-10-20

**Authors:** Huei Chu Kao, Chiuhsiang Joe Lin, Yung Hui Lee, Su Huang Chen

**Affiliations:** 1 Graduate Institute of Management, National Taiwan University of Science and Technology, Taipei, Taiwan, ROC; 2 Department of Industrial Management, National Taiwan University of Science and Technology, Taipei, Taiwan, ROC; University of Rome Foro Italico, ITALY

## Abstract

The purpose of this study was to explore the effects of direction of exertion (DOE) (pushing, pulling), path (walking in a straight line, turning left, walking uphill), and load placement (LP) (the 18 blocks were indicated by X, Y and Z axis; there were 3 levels on the X axis, 2 levels on the Y axis, and 3 levels on the Z axis) on muscle activity and ratings of perceived exertion in nursing cart pushing and pulling tasks. Ten participants who were female students and not experienced nurses were recruited to participate in the experiment. Each participant performed 108 experimental trials in the study, consisting of 2 directions of exertion (push and pull), 3 paths, and 18 load placements (indicated by X, Y and Z axes). A 23kg load was placed into one load placement. The dependent variables were electromyographic (EMG) data of four muscles collected bilaterally as follows: Left (L) and right (R) trapezius (TR), flexor digitorum superficialis (FDS), extensor digitorum (ED), and erector spinae (ES) and subjective ratings of perceived exertion (RPE). Split-split-plot ANOVA was conducted to analyze significant differences between DOE, path, and LP in the EMG and RPE data. Pulling cart tasks produced a significantly higher activation of the muscles (RTR:54.4%, LTR:50.3%, LFDS:57.0%, LED:63.4%, RES:40.7%, LES:36.7%) than pushing cart tasks (RTR:42.4%, LTR:35.1%, LFDS:32.3%, LED:55.1%, RES:33.3%, LES:32.1%). A significantly greater perceived exertion was found in pulling cart tasks than pushing cart tasks. Significantly higher activation of all muscles and perceived exertion were observed for walking uphill than walking in a straight line and turning left. Significantly lower muscle activity of all muscles and subject ratings were observed for the central position on the X axis, the bottom position on the Y axis, and the posterior position on the Z axis. These findings suggest that nursing staff should adopt forward pushing when moving a nursing cart, instead of backward pulling, and that uphill paths should be avoided in the design of work environments. In terms of distribution of the load in a nursing cart, heavier materials should be positioned at bottom of the cabinet, centered on the horizontal plane and close to the handle, to reduce the physical load of the nursing staff.

## Introduction

Many studies have pointed out that nurses are at high risk of musculoskeletal disorders (MSDs) [1–6]. Nursing is heavy physical work that often requires bending of the back, turning patients, pushing and pulling medical carts and hospital beds, sometimes even carrying heavy loads [7, 8]. Studies have found that the main causes of musculoskeletal injuries are overexertion and cumulative load [9]. In addition to processing information, nurses also need to handle patient care, examinations, and medicine. Considering the feasibility of clinical practices, nursing carts with computers installed have been developed to assist nursing staff in hospitals and clinics. Nursing staff can move such carts freely to take care of the patients, freeing them from a fixed nursing station. The nursing cart can be used to reduce travel between the nursing station and the patient, thereby making nursing more efficient. However, research on nursing carts is rather rare. The operations of cart pushing and pulling belong to manual material handling (MMH). Results and recommendations from pushing and pulling research in MMH literature can be used to help the design of nursing cart handling tasks and the cart design itself.

MMH activities include lifting, lowering, carrying, holding, pushing, and pulling [10], and such activities are the main causes of MSDs, especially those of the arm, neck, shoulder, and lower back [11]. To reduce the risk of musculoskeletal injuries, operational equipment such as carts, fork-lift trucks, and conveyers are used to assist with manual handling tasks [12]. Although these auxiliary equipment can facilitate MMH, they increase the number of pushing and pulling tasks [13–16]. An investigation of NIOSH (1981) pointed that lower back pain (LBP) was related to pushing and pulling and comprised about 20% of overexertion injuries in the USA [17]. Klein et al. (1984) indicated that 9% of lower back pain was connected with pushing and pulling tasks [18]. Some studies have also shown that 9–18% of LBP is correlated to pushing and pulling tasks [10, 19–21]. However, a more recent study by Roffey et al. (2010) investigated causal relationships between occupational pushing/pulling and low back pain in 13 studies and found that no study has satisfied the criteria of epidemiologic approach in setting a causal relationship between pushing/pulling and low back pain. The study suggested that at the moment no consistent evidence has been presented to support causation for occupational pushing/pulling and low back pain [22].

Despite the survey results uncovered by Roffey et al. (2010), Hoozemans et al. (2014) indicated that there was strong evidence for a causal association between pushing/pulling and upper extremity symptoms [23]. Several other studies indicated that pushing and pulling tasks could cause musculoskeletal injuries of the lower back, shoulders, and upper limbs [24, 25]. A few other studies have also found that pushing and pulling tasks were related to musculoskeletal injures of the upper body, especially the shoulders, neck, and upper limbs [9, 26–27]. In addition, a self-discomfort questionnaire also found that the lower back and upper limbs experienced discomfort during performing pushing and pulling tasks. However, the factors causing such discomfort have not been fully explained [28, 29].

Jung et al. (2005) indicated four situations that define the direction of motion. When a cart is located in front of the operator, the situations are (1) forward pushing and (2) backward pulling, and when the cart is behind the operator, the situations are (3) forward pulling and (4) backward pushing [15]. Some pushing and pulling biomechanical studies have been reviewed [9, 30] and more detailed biomechanical models were suggested to accurately address the forces and moments at the joints and muscles. Lin et al. (2010) explored the effects of using a four-wheeled manual guided vehicle on the flexor digitorum superficialis, anterior deltoid, trapezius and erector spinae. The result showed that the bilateral muscle activities of flexor digitorum superficialis, anterior deltoid, trapezius and erector spinae in the pulling tasks were significantly higher than those in the pushing tasks [31]. Other studies of four-wheeled carts exploring the influence of pushing and pulling tasks on the erector spinae, have reported that a pulling task causes higher L5/S1 compressive force and torque [9, 32–33]. Chen et al. (2014) reported that a pulling task with a one-wheeled wheelbarrow induced a higher muscle activity of erector spinae than a pushing task [34]. It must be noted that this study was done with a one-wheeled wheelbarrow and the results from different types of carts should be compared with care due to their different mechanisms.

Some researchers have noted that the center of mass (COM) of a cart can affect the operator’s work performance [34, 35]. Kingma et al. (2003) studied the COM and handle locations of a two-wheeled dust-cart to examine the effects on hand force and joint load [35]. The dust-cart was divided into nine COMs in accordance with the sagittal plane. Results showed that the COM and handle location affect hand force and joint load in pushing and pulling a two-wheeled dust-cart. They found that the load on the back was greater at greater distances from COM to participant, and that the load on the shoulder and elbow was greater at smaller distances from COM to participant. These results suggested that two-wheeled dust-carts should be designed such that the location of the COM is closer to the axis of the wheels to reduce the joint loading and increase the control stability. Chen et al. (2014) studied the effects of nine different load placements on muscles in the back during the operation of a one-wheeled wheelbarrow. Results showed that the closer the weight was to the participant, the greater the load was on the back. The least load was found with the weight placed in the front of the bin [34]. It is worth noting that the mechanics in handling two- and one-wheeled carts may be totally different and might not be comparable to the mechanics in handling four-wheeled carts and should not be treated as the same things. There were very few studies in handling four-wheeled carts in the past, however, performing four-wheeled carts in the industry are common. Therefore, it is worth to study the issues related to performing four-wheeled carts.

According to the studies of various carts above, the COM, force direction, and path are important factors during cart operation. Due to the large number of hospitals using various types of digital equipment to help nursing staff, and the concomitant increase in the weight of the nursing cart, this study was conducted to evaluate factors in cart operation and provide recommendations for nursing cart use.

Nursing carts usually include a cart, a computer, a stand, a battery, a power line, and nursing materials. These components in the nursing cart's configuration will affect the COM of the cart, but no research has explored this issue. Therefore, the purpose of this study was to explore the effects of direction of exertion (DOE) (pushing, pulling), path (walking in a straight line, turning left, walking uphill), and load placement (LP) (the 18 blocks were indicated by X, Y and Z axis. There were 3 levels on the X axis, 2 levels on the Y axis, and 3 levels on the Z axis.) on muscle activity and ratings of perceived exertion (RPE) in nursing cart pushing and pulling tasks.

## Methods

### Participants

Students at National Taiwan University of Science and Technology were recruited to participate in the experiment. Each participant had to complete a health questionnaire before any tests to evaluate their qualifications. Participants were excluded from the study if they reported any pain, injuries, or history of musculoskeletal disorders. Ten healthy female college students qualified and were paid to participate in the experiment. Participants had no prior experience in performing pushing and pulling nursing carts so that they did not possess any prior impression about the tasks or carts. Each participant had to practice performing pushing and pulling tasks before starting the experiment. Participants provided their written informed consent to participate in this study and were familiarized with the experimental process. The study was approved by the Research Ethics Committee of National Taiwan University. The mean age, height, and weight were 22 years (SD = 2.24), 161.7 cm (SD = 2.65), and 52.9 kg (SD = 4.40), respectively. Detailed information on and the anthropometry of all participants are shown in Table 1.

**Table 1:** Data of all participants (n = 10).

characteristic	mean	SD	MAX	min
age (yrs)	22.0	2.24	27.0	19.0
body height (cm)	161.7	2.65	165.0	158.0
body mass (kg)	52.9	4.40	61.0	47.0
shoulder height (cm)	132.9	2.87	138.5	129.5
elbow height (cm)	101.7	2.08	105.0	99.0
knuckle height (cm)	71.8	2.87	76.0	68.0
knee height (cm)	45.5	3.52	52.5	41.5

### Apparatus

#### Electromyography

Muscle activation was recorded with surface electromyography (EMG). The wireless EMG system (TELEMYO 2400T) was produced by the Noraxon Company. During the pushing and pulling activity, several muscles could be involved including trapezius, anterior deltoid, flexor digitorum, erector spinae, brachioradialis, and latissimus dorsi [31, 34, 36] Due to the limit in the availability of the number of channels, trapezius (TR), flexor digitorum superficialis (FDS), extensor digitorum (ED), and erector spinae (ES) were selected in the study. These muscles account for the exertion needed for the cart tasks. EMG data of four muscles were collected bilaterally. For the TR muscle, the electrode placements were halfway on the line from the acromion to the spine on vertebra C7. The position of the electrode for the extensor digitorum (ED) was determined to be on the line formed by the lateral epicondyle and midpoint of the two styloids and the electrode was placed on the superior-ulnar side of the forearm one third of the forearm distance distal to the lateral epicondyle. For the FDS muscle, the electrode was placed midway along the palmar side of the forearm where the muscle contraction could be felt. For the ES muscle, the electrode placements were at two fingers’ width laterally at the L3 level. A reference electrode was placed on C7 were placed according to the method described in Perotto (1994) [37]. The bipolar surface electrodes (Ag/AgCl) were placed on these muscles and the EMG signals were recorded during pushing and pulling. The sampling rate was 1000 Hz with high-pass filtering at 6 Hz and low-pass filtering at 600 Hz, integrated using the Root-Mean-Square (RMS) (75 ms) method. All the EMG data were processed and analyzed with MyoResearch software. Before the experiment, the maximal voluntary contraction (MVC) of these muscles was measured with reference to Hislop and Montgomery (2007) [38], respectively. The RMS of the EMG values during pushing and pulling was normalized to the MVC so that the data could be expressed as %MVC.

#### Ratings of perceived exertion

The Borg CR-10 scale is a tool for quantifying subjective response. The use of the Borg CR-10 is quite broad, including human factors evaluation, difficulty evaluation, and other perceived feelings, such as taste or vision [39]. Immediately following the trial’s completion, participants were requested to rate their perceived exertion with the score from 0 that meant nothing at all to a score of 10 that meant impossible (see Table 2).

**Table 2:** Borg CR-10 Ratings of perceived exertion.

Rating	Definition
0	Nothing at all
0.5	Very, very easy
1	Very easy
2	Easy
3	Moderate
4	Somewhat hard
5	Hard
6	
7	Very hard
8	
9	Very, very hard
10	Impossible

#### Cart

**Fig 1:**
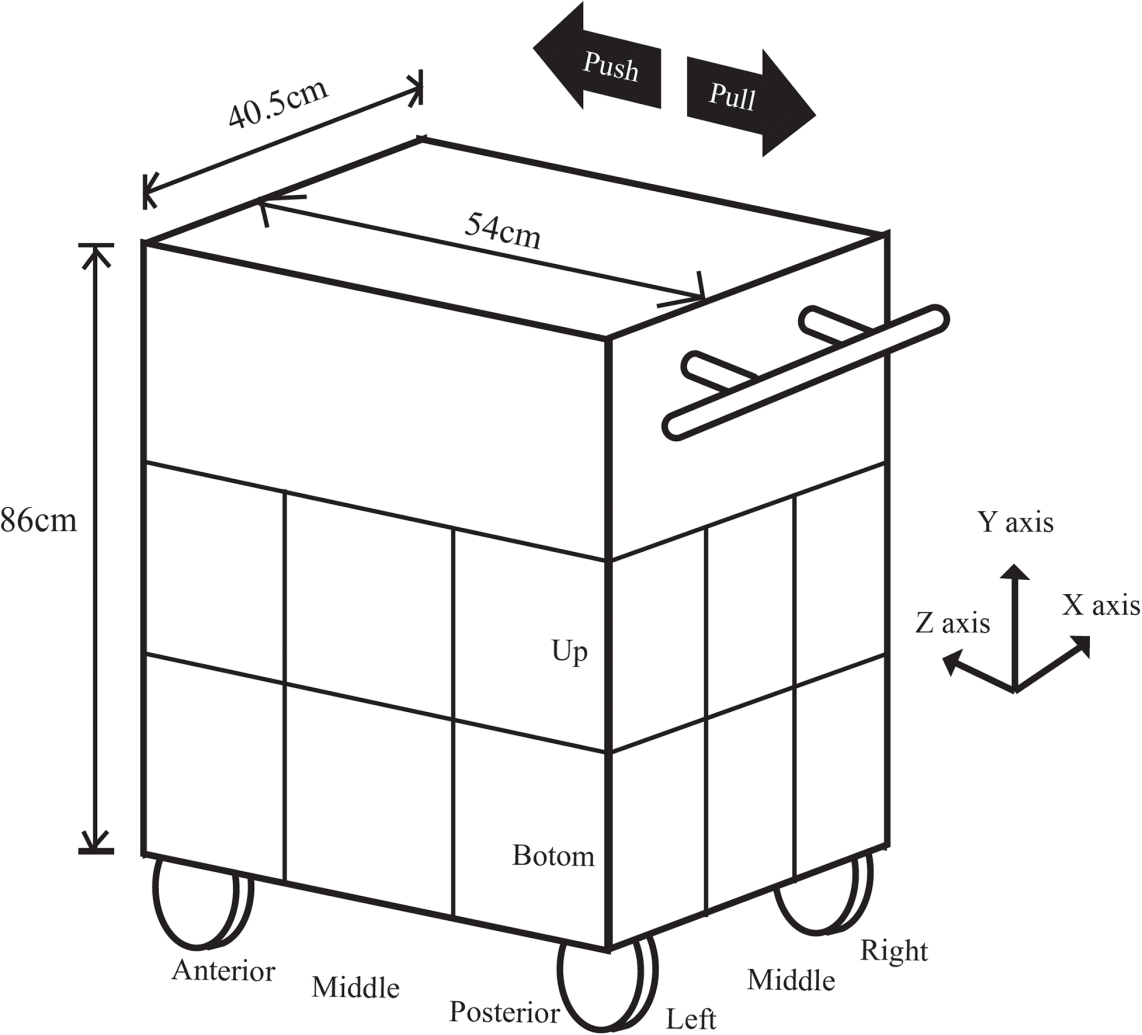
The nursing cart used in the study with the varied load placements indicated by X, Y, and Z axes. The force directions are shown by the arrows.

The cart dimension was shown in Fig 1. The four hard rubber wheels were rotatable. Three wood boxes (length x width x depth: 40.5 x 54 x 86 cm) were placed on the cart. The bottom and middle boxes were divided into 9 blocks. The 18 blocks were indicated by X, Y and Z axes. There were 3 levels on the X axis, 2 levels on the Y axis, and 3 levels on the Z axis. A load of about 23 kg was placed into a single block of a wooden box. Three wood boxes, wheels and other parts were weighed to be 52 kg. The total weight was 75 kg. The dimensions, the wheels and the weight were comparable to the carts used by nurses.

#### Path

**Fig 2:**
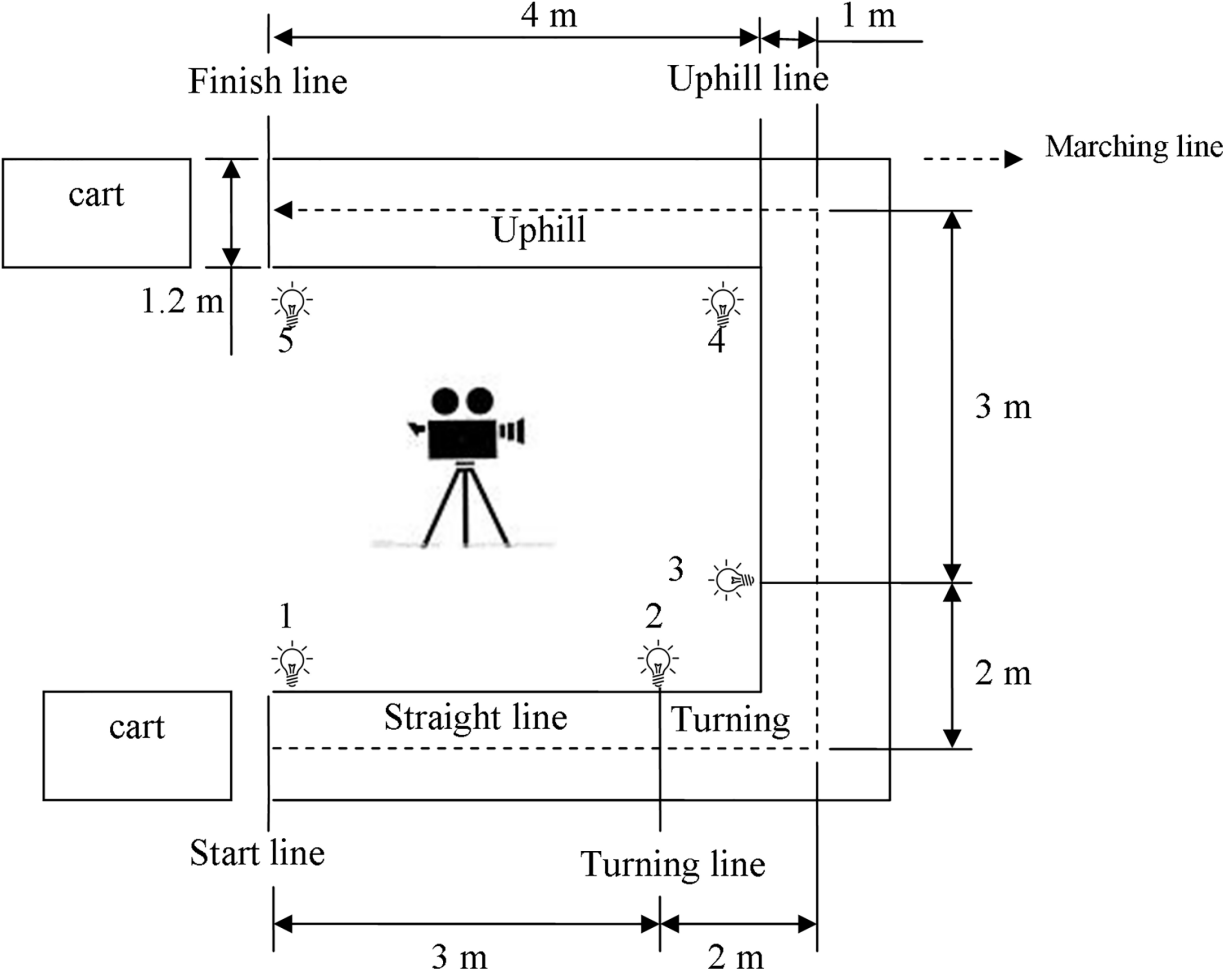
Push/pull track diagram with motion phase sequence.

The path in this study was made of wood. The coefficient of friction between path and wheel was 0.5, as measured by Brungraber Mark II. The width of the path was 120cm, and the length of a straight line was 500cm. The length of the uphill was 400cm, and the height was 33cm, creating an uphill slope of 4.7°. Each start line and finish line of the paths was marked by colored tape, and lights were used to mark time points for processing with a video camera (see Fig 2). The path was designed to simulate the cart tasks encountered in daily nursing work, which may include turning, flat and straight pushing or pulling, and uphill pushing or pulling.

### Experimental procedure

In the beginning, participants were familiarized with the experimental procedures and performed stretching exercises for at least ten minutes before beginning the experiment. During the experimental trials, participants were required to wear light clothing and shoes with rubber soles to obtain a static coefficient of friction of at least 0.5 between the shoe sole and floor, as generally recommended for manual materials handling [40]. Each participant’s height, body weight, shoulder height, elbow height, knuckle height, and knee height were measured. The MVC of the muscle groups on the left and right sides were then collected. Each participant performed the various testing combinations in a random order and began pushing/pulling a cart at the sound of a whistle. Testing combinations and experimental factors are described below in the experimental design section. Before performing the test, all participants were given the following instructions: “When you hear the whistle, grasp the handles within about 1s, and then start to push (pull) the cart straight for a distance of 3m, followed by a 90° turn, followed by continuing straight for 3m, followed by a distance of 4m uphill (slope: 4.7°) to the finish line.” The start and finish lines of each path were marked on the floor with colored tape (see Fig 2). All participants pushed/pulled the cart in a natural posture and at a controlled pace (80 steps per minute) set by a metronome. Between trials, participants took a three-minute rest. A ten-minute rest was scheduled after completion of the nine trials. The data on muscle EMGs and RPE were collected during the pushing/pulling tasks.

### Data analysis

A split-split-plot design was used in the study. Significance was set at the p<0.05 level. The independent variables were DOE (pushing, pulling), path (walking in a straight line, turning left, walking uphill) and load placement (LP). Each placement was plotted on X, Y, and Z axes. The X axis had 3 levels; the Y axis, 2 levels; and the Z axis, 3 levels. Whole-plot was exertion direction, and block was Participants. Tukey post hoc analyses identified the position of various significant differences. The dependent variables were RPE and the EMG of both sides of the trapezius (TR), flexor digitorum superficialis (FDS), extensor digitorum (ED), and erector spinae (ES). Each participant performed 108 trials of the experiment.

## Result

### The effects of direction of exertion

The experiment was performed as planned and the data were collected without missing data. Tables 3 and 4 presented the mean EMG and mean RPE under the effect levels of each independent variable, respectively. As shown in Table 3, the muscle activity of the pulling task was higher than that of the pushing task (p<0.05), which was about 36.7%-63.4%. The greatest difference in muscle activity (~25%) between pushing and pulling was in the LFDS. The smallest difference (4%) was in the LES. The RPE results indicated that the pulling tasks required more effort than the pushing tasks (pull: 3.409 vs. push: 2.652)(p<0.001).

**Table 3:** Muscle EMG (%MVC) for each independent variable.

	RTR	LTR	RFDS	LFDS	RED	LED	RES	LES
**DOE**								
Push	**42.4**	**35.1**	44.5	**32.3**	51.9	**55.1**	**33.3**	**32.1**
Pull	**54.4**	**50.3**	49.9	**57.0**	49.4	**63.4**	**40.7**	**36.7**
**Path**								
Straight line	**36.7** ^**a**^	**31.9** ^**a**^	**36.0** ^**a**^	**36.3** ^**a**^	**42.1** ^**a**^	**51.6** ^**a**^	**32.2** ^**a**^	**29.0** ^**a**^
Turning left	**49.3** ^**b**^	**44.2** ^**b**^	**49.9** ^**b**^	**46.2** ^**b**^	**50.9** ^**b**^	**57.6** ^**b**^	**37.4** ^**b**^	**33.7** ^**b**^
Walking uphill	**59.2** ^**c**^	**52.1** ^**c**^	**55.7** ^**c**^	**51.5** ^**c**^	**59.0** ^**c**^	**68.7** ^**c**^	**41.4** ^**c**^	**40.5** ^**c**^
**X**								
Left	**51.8** ^**b**^	**45.6** ^**b**^	**51.1** ^**b**^	**46.7** ^**b**^	**53.1** ^**b**^	**61.6** ^**b**^	**40.1** ^**b**^	**37.3** ^**b**^
Middle	**41.8** ^**a**^	**35.8** ^**a**^	**40.9** ^**a**^	**38.3** ^**a**^	**43.9** ^**a**^	**53.5** ^**a**^	**30.1** ^**a**^	**27.5** ^**a**^
Right	**51.6** ^**b**^	**46.7** ^**b**^	**49.6** ^**b**^	**48.9** ^**c**^	**55.1** ^**b**^	**62.7** ^**b**^	**40.9** ^**b**^	**38.4** ^**b**^
**Y**								
Low	**45.6**	**40.1**	**44.2**	**41.7**	**48.4**	**56.9**	**33.8**	**32.2**
High	**51.3**	**45.3**	**50.2**	**47.7**	**53.0**	**61.6**	**40.2**	**36.6**
**Z**								
Posterior	**44.6** ^**a**^	**38.4** ^**a**^	**43.1** ^**a**^	**40.7** ^**a**^	**46.7** ^**a**^	**56.4** ^**a**^	**34.0** ^**a**^	**30.7** ^**a**^
Middle	**49.7** ^**b**^	**44.1** ^**b**^	**48.6** ^**b**^	**46.1** ^**b**^	**52.7** ^**b**^	**60.6** ^**b**^	**37.9** ^**b**^	**35.2** ^**b**^
Anterior	**50.9** ^**b**^	**45.6** ^**b**^	**50.0** ^**b**^	**47.2** ^**b**^	**52.7** ^**b**^	**60.8** ^**b**^	**39.1** ^**b**^	**37.3** ^**b**^

^a, b, c^: Tukey HSD grouping code. R, right side; L, left side; TR, trapezius; FDS, flexor digitorum superficialis; ED, extensor digitorum; ES, erector spinae; DOE, direction of exertion. Bold indicates significant differences between levels of a factor for that measure.

**Table 4:** RPE (scores) for each independent variable.

	Mean (SD)	Range
**DOE**		
Push	**2.652 (1.737)**	1–10
Pull	**3.409 (1.662)**	1–10
**Path**		
Straight	**1.727** ^a^ **(0.781)**	1–4
Turning	**2.737** ^b^ **(1.115)**	1–7
Uphill	**4.626** ^c^ **(1.705)**	2–10
**X**		
Left	**2.907** ^a^ **(1.646)**	1–9
Middle	**2.963** ^a^ **(1.694)**	1–10
Right	**3.220** ^b^ **(1.863)**	1–10
**Y**		
High	**3.133(1.735)**	1–10
Low	**2.927(1.741)**	1–10
**Z**		
Posterior	**2.896** ^a^ **(1.683)**	1–9
Middle	**3.052** ^a^ ^b^ **(1.719)**	1–10
Anterior	**3.142** ^b^ **(1.812)**	1–10

^a, b, c^: Tukey HSD grouping code. DOE, direction of exertion; RPE, ratings of perceived exertion.

Bold indicates significant differences between levels of a factor for that measure.

### The effects of path

**Fig 3:**
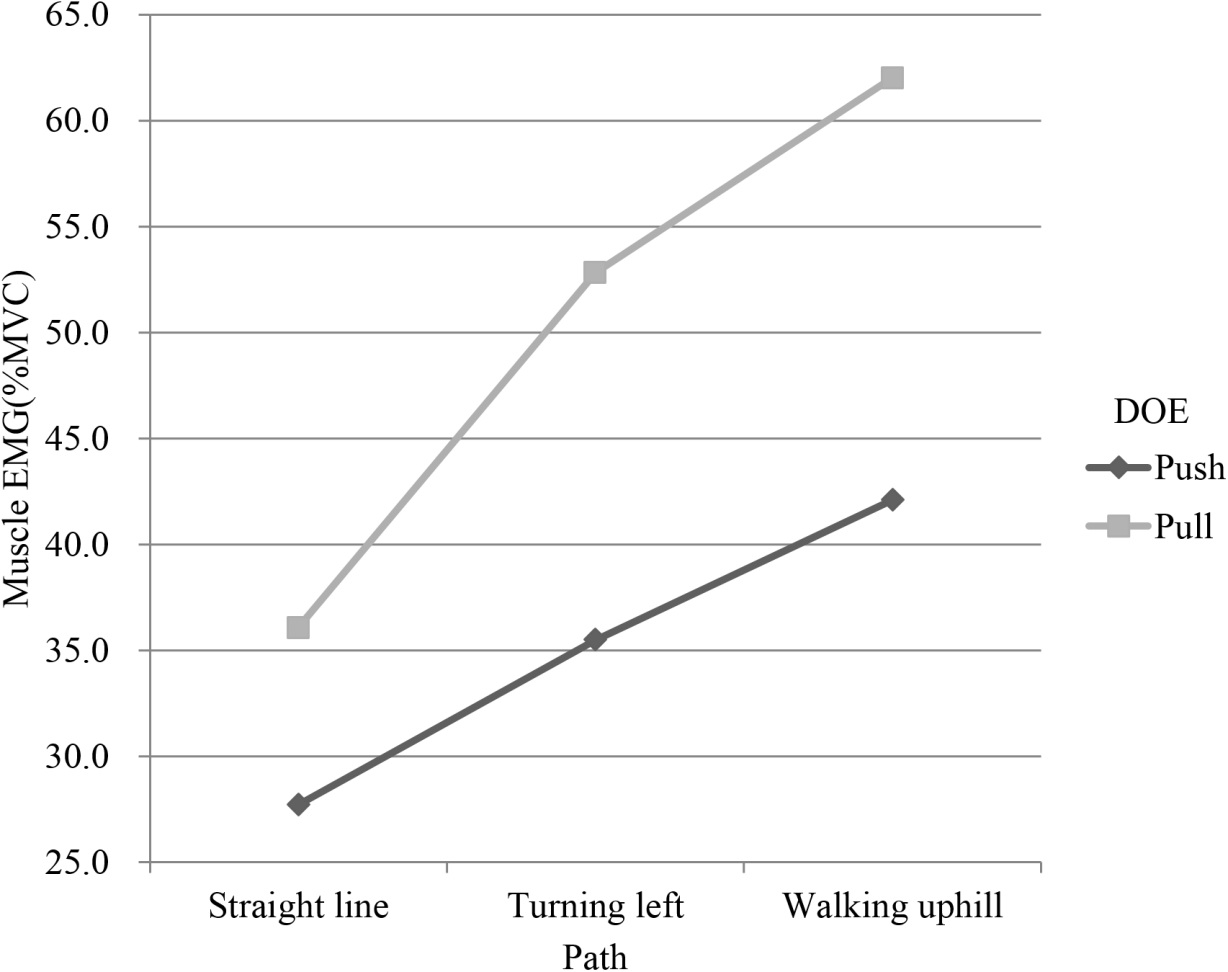
Interaction plot of path and direction of exertion (DOE) in left trapezius (LTR).

**Fig 4:**
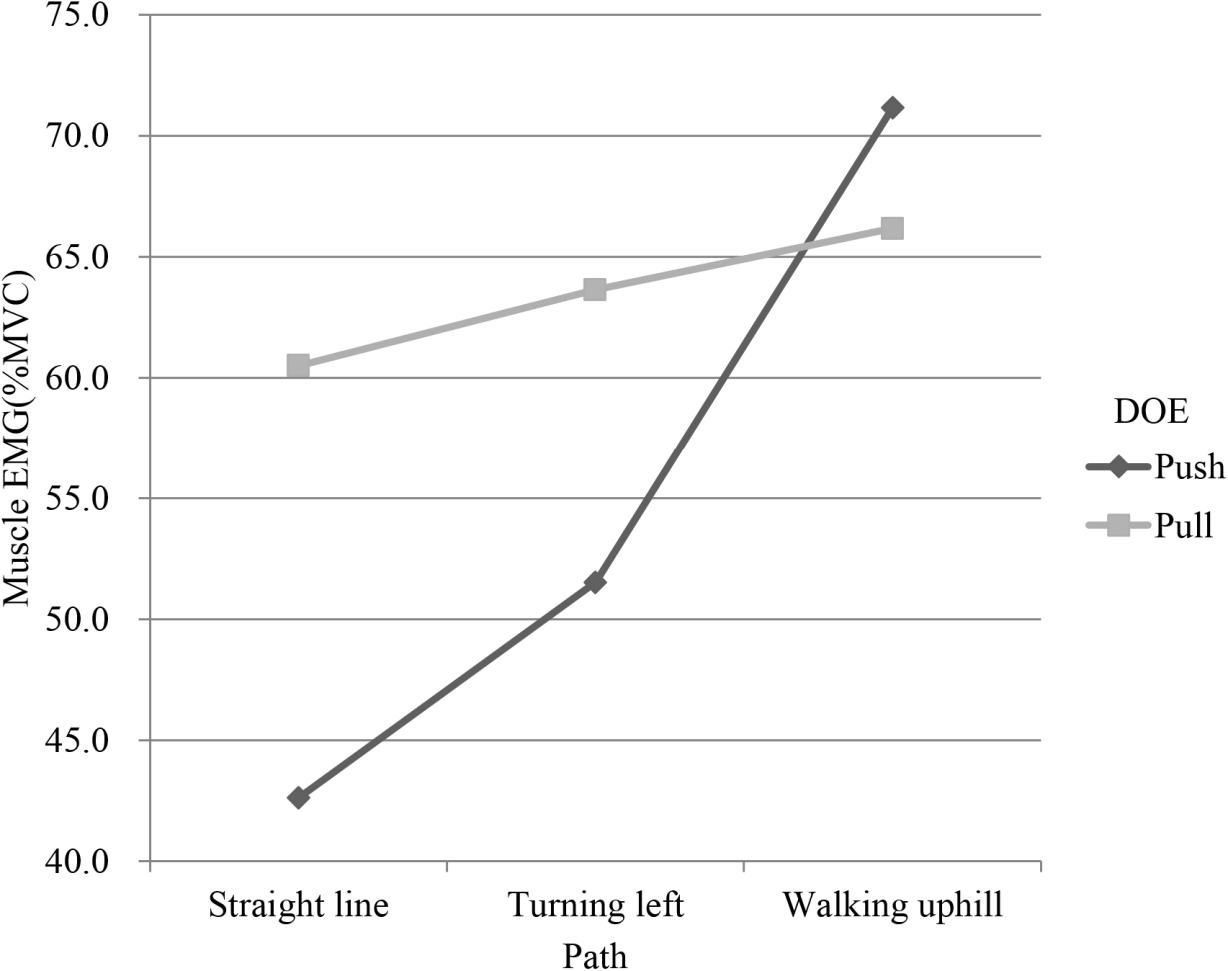
Interaction plot of path and direction of exertion(DOE) in left extensor digitorum (LED).

**Fig 5:**
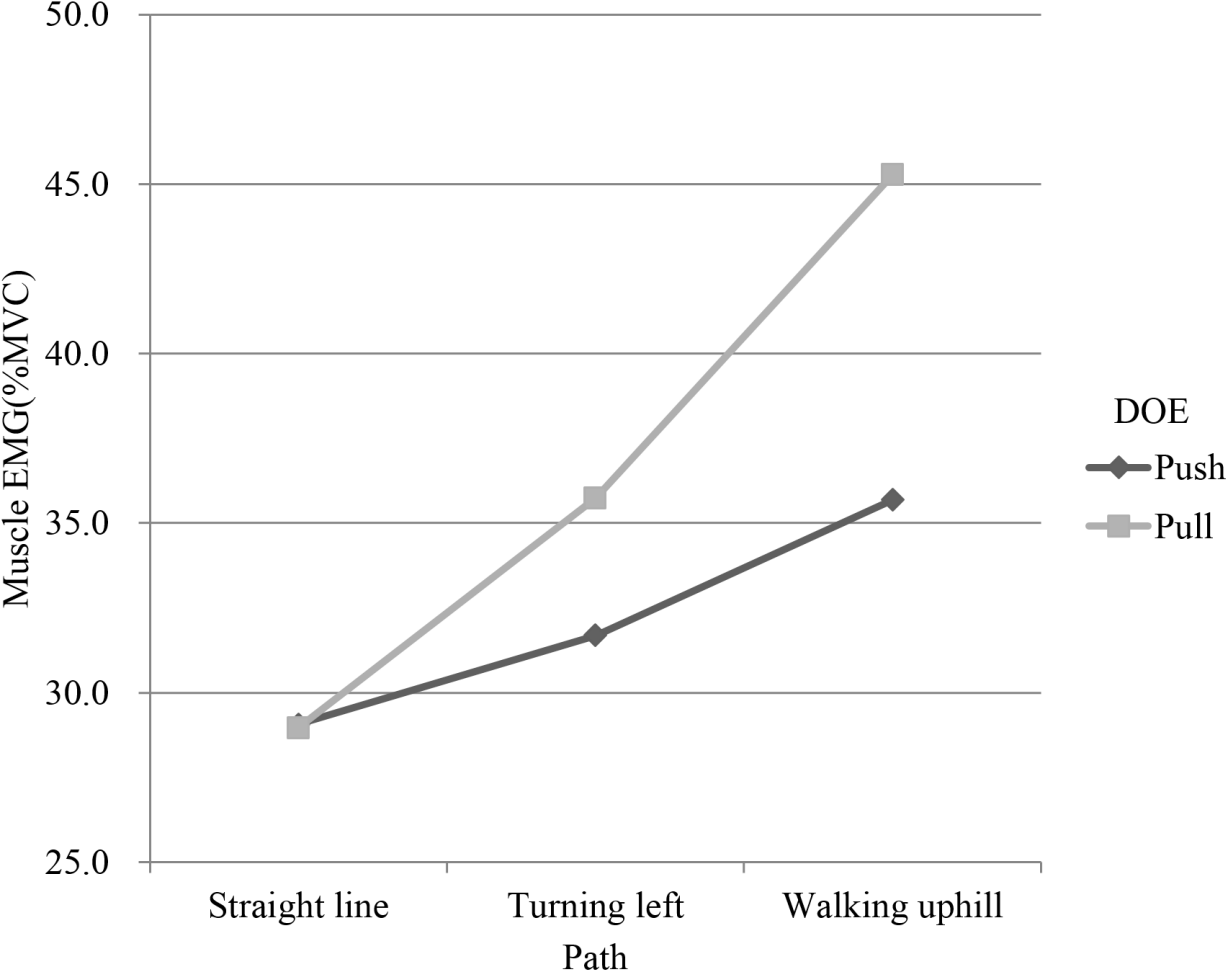
Interaction plot of path and direction of exertion(DOE) in left erector spinae (LES).

Path had significant effects on EMG and RPE (p<0.001). Tukey test showed that the path was divided into 3 groups (Tables 3 and 4). The first group was the straight line, the second was the turning path, and the last was the uphill path. The bilateral EMGs of uphill were higher than those of turning and straight line. LTR, LED and LES had an interaction between path and DOE (Figs 3–5). In addition, as shown in Table 4, uphill required more perceived effort than straight line and turning.

### The effects of load placement

The EMG of all muscles varied significantly with LP on the X, Y and Z axes (p<0.001). Tukey test showed that on the X axis, all muscle activity for the left and right LPs was higher than that for the middle LP. On the Y axis, the bottom LP had lower EMG for all muscles. On the Z axis, when the LP was close to the participant, all muscle activity was lower (Table 3). A similar result was also found from the RPE (Table 4).

### Summary of ANOVA results

Tables 5–7 presented the statistical test results for EMG of all the muscles. Except for the RFDS and RED, all the muscles were affected by DOE significantly (p<0.05). All muscle activity was influenced by the various paths. Interactions were found between path and DOE in LTR, LED, and LES. Finally, LP (X, Y, and Z axis) affected all muscle activities significantly (p<0.001). There were interactions between DOE and X axis in RTR and LFDS. There was an interaction between DOE and Z axis in RFDS and LES. Tables 8–10 show the effects of different DOEs, paths and LPs on the RPE. The results showed that RPE was significantly affected by all independent variables (p<0.001). In addition, interactions were found between X and DOE; Y and DOE; and X, DOE and path.

**Table 5:** Summary of P value on X axis of muscle activity in ANOVA results.

Source	df	RTR	LTR	RFDS	LFDS	RED	LED	RES	LES
DOE	1	0.003**	0.004**	0.306	0.000***	0.448	0.026*	0.003**	0.032*
Path	2	0.000***	0.000***	0.000***	0.000***	0.000***	0.000***	0.000***	0.000***
DOE*Path	2	0.447	0.019*	0.052	0.743	0.779	0.000***	0.565	0.004**
X	2	0.000***	0.000***	0.000***	0.000***	0.000***	0.000***	0.000***	0.000***
DOE*X	2	0.006**	0.698	0.817	0.020*	0.834	0.217	0.191	0.059
Path*X	4	0.823	0.767	0.349	0.423	0.865	0.087	0.916	0.087
DOE*Path*X	4	0.112	0.634	0.162	0.644	0.081	0.565	0.946	0.500

*, Significant at p<0.05;

**, Significant at p<0.01;

***, Significant at p<0.001.

R, right side; L, left side; TR, trapezius; FDS, flexor digitorum superficialis; ED, extensor digitorum; ES, erector spinae; DOE, direction of exertion.

**Table 6:** Summary of P value on Y axis of muscle activity in ANOVA results.

Source	df	RTR	LTR	RFDS	LFDS	RED	LED	RES	LES
DOE	1	0.003**	0.004**	0.306	0.000***	0.448	0.026*	0.003**	0.032*
Path	2	0.000***	0.000***	0.000***	0.000***	0.000***	0.000***	0.000***	0.000***
DOE*Path	2	0.447	0.019*	0.052	0.743	0.779	0.000***	0.565	0.004**
Y	1	0.000***	0.000***	0.000***	0.000***	0.000***	0.000***	0.000***	0.000***
DOE*Y	1	0.585	0.911	0.296	0.912	0.054	0.365	0.733	0.607
Path*Y	2	0.975	0.486	0.651	0.593	0.217	0.116	0.252	0.124
DOE*Path*Y	2	0.295	0.642	0.282	0.135	0.758	0.320	0.075	0.841

*, Significant at p<0.05;

**, Significant at p<0.01;

***, Significant at p<0.001.

R, right side; L, left side; TR, trapezius; FDS, flexor digitorum superficialis; ED, extensor digitorum; ES, erector spinae; DOE, direction of exertion.

**Table 7:** Summary of P value on Z axis of muscle activity in ANOVA results.

Source	df	RTR	LTR	RFDS	LFDS	RED	LED	RES	LES
DOE	1	0.003**	0.004**	0.306	0.000***	0.448	0.026*	0.003**	0.032*
Path	2	0.000***	0.000***	0.000***	0.000***	0.000***	0.000***	0.000***	0.000***
DOE*Path	2	0.447	0.019*	0.052	0.743	0.779	0.000***	0.565	0.004**
Z	2	0.000***	0.000***	0.000***	0.000***	0.000***	0.000***	0.000***	0.000***
DOE*Z	2	0.574	0.614	0.034*	0.054	0.872	0.779	0.383	0.046*
Path*Z	4	0.337	0.612	0.703	0.447	0.790	0.581	0.440	0.486
DOE*Path*Z	4	0.657	0.028*	0.814	0.595	0.089	0.610	0.990	0.208

*, Significant at p<0.05;

**, Significant at p<0.01;

***, Significant at p<0.001.

R, right side; L, left side; TR, trapezius; FDS, flexor digitorum superficialis; ED, extensor digitorum; ES, erector spinae; DOE, direction of exertion.

**Table 8:** Summary of P value on X axis of RPE in ANOVA results.

Source	df	RPE
DOE	1	0.000***
Path	2	0.000***
DOE*Path	2	0.630
X	2	0.000***
DOE*X	2	0.000***
Path*X	4	0.394
DOE*Path*X	4	0.028*

*, Significant at p<0.05;

***, Significant at p<0.001.

DOE, direction of exertion; RPE, ratings of perceived exertion.

**Table 9:** Summary of P value on Y axis of RPE in ANOVA results.

Source	df	RPE
DOE	1	0.000***
Path	2	0.000***
DOE*Path	2	0.630
Y	1	0.000***
DOE*Y	1	0.195
Path*Y	2	0.000***
DOE*Path*Y	2	0.129

***, Significant at p<0.001.

DOE, direction of exertion; RPE, ratings of perceived exertion.

**Table 10:** Summary of P value on Z axis of RPE in ANOVA results.

Source	df	RPE
DOE	1	0.000***
Path	2	0.000***
DOE*Path	2	0.630
Z	2	0.001***
DOE*Z	2	0.939
Path*Z	4	0.316
DOE*Path*Z	4	0.997

***, Significant at p<0.001.

DOE, direction of exertion; RPE, ratings of perceived exertion.

## Discussion

### Influence of direction of exertion

In this study, participants pushed/pulled a simulated nursing cart with various load placements along several paths (walking in a straight line, turning left, walking uphill), and the muscle activity and subjective responses were measured under these experimental combinations. The results showed that muscle activity was lower in the pushing task than in the pulling task. This difference suggests that one way to reduce all muscle loads when operating a nursing cart is to push the cart forward. The RPE results suggested the same finding (Table 4). Previous studies about pushing/pulling tasks indicated that cart pushing tasks require less force than cart pulling tasks [41] and incur smaller back loads [20, 26, 31, 33]. Al-Eisawi et al. (1999a) reported that the push force to operate a cart of the same weight is, on average, 93.5% of the pull force [42]. Nijenhuis and Roseboom (1987) also found that mean heart rates were lower during pushing tasks than during pulling tasks [43]. In addition, pushing tasks incur lower loads on the back than pulling tasks [20, 26, 44]. Finally, Lin et al. (2010) reported that muscle activity in the FDS, TR and ES was lower during pushing tasks than during pulling tasks [31]. Therefore, it is suggested that it is better to push a nursing cart than to pull one.

### Influence of path

Most studies on pushing and pulling have explored only the effects of a straight path [15, 45]. However, such a narrow focus cannot accurately represent the real work environment. Lin et al. (2010) designed a pushing and pulling experiment including both straight and turning paths [31]. However, their results did not compare the differences in the various routes. That study compared three various paths in pushing and pulling tasks. The results showed that uphill tasks produced the greatest muscle activity on all the muscles. Subjective evaluation also suggested that uphill tasks required the most effort, and that the task requiring the least effort was the straight line. Some studies have analyzed the effect of gradient on pushing force and found that a higher gradient requires greater pushing force [46, 47]. The RPE scores in these studies were also higher for higher gradients. These findings support the assumption that uphill tasks require more physiological energy. In addition, most participants are inclined to bend the trunk forward to use their body weight to increase the pushing force, especially during pushing tasks. Moorea et al. (2013) explored the influence of gradient on kinematics and found that the larger the gradient was, the greater the angle of trunk flex was [48]. Therefore, an uphill design will increase a participant’s physiological and psychological load in pushing and pulling tasks. From the above, it is clear that a work environment for nursing staff should be designed with few uphill slopes.

It is worth noting that the interactions between path and DOE on LTR, LED, and LES muscular activity were significant (Figs 3–5). The muscular activity of the forearm was of large difference between push and pull on the straight and turn but the difference decreased on uphill and the uphill had a higher muscular activity than the straight and turn. During uphill, it could be possible that much more force is needed and the forearm force must be combined with other body part such as the back to complete the task, reducing the role of forearm in the uphill task. On the flat floor, however, forearm plays a major role in manipulating the cart and the exertion for the push and pull are thus quite different. This can be supported by the interaction on the muscular activity of the back which showed an opposite pattern where the straight line had a small difference but the turn and uphill had an enlarged difference and was higher. This shows that different part of the body had been recruited and their utilization was also different for pushing and pulling the cart on different paths.

### Influence of load placement

In general, the nursing cart has a computer, a stand, a battery, a power line, and nursing materials. Although the ideal load distribution is to spread the load on the nursing cart equally, it is difficult to do so in practice. Thus, this study analyzed the effects of various LPs on the bilateral EMG of TR, FDS, ED, ES and RPE. The results showed that the lowest EMG and RPE appeared when the load was placed in the middle of the X axis, at the bottom of the Y axis, and closest to the participant on the Z axis. That is, when participants pushed/pulled a four-wheeled cart in which the load was placed at the anterior part of the bin (far from the participant), participants tended to perform the push/pull with a higher muscle activity than when the load was placed at the posterior part of the bin (close to the participant). This can be explained by the following maneuvering strategy. If the posterior wheels are considered as the fulcrum, when the load was at the anterior part, the arm of effort (hand to posterior wheel) was much shorter than the resisting arm (load to posterior wheel). Any disturbance to the cart due to the uneven floor would need a higher force to maintain it in the intended direction. Therefore, the arms had to exert more to keep the cart in its intended path under disturbance. In this strategy, to turn the cart would also need a larger force from the arms. We observed that the participant used this strategy to turn the cart, that is, the head of the cart moved sideways prior to the person moving laterally during the turn. In fact, during the turn, one can adopt the other strategy using the anterior wheels as the fulcrum. An opposite effect would occur and the person would have to move laterally first to turn the cart. Our participants did not follow this strategy because one would have to move to the left and then move to the right (zigzag) to control the forward direction of the cart.

### Limitations of the study

In this study, one limitation was the relatively small number of participants, and their characteristics. All were female university students, not actual nurses. It is recommended that a broader sample including males and participants of other ages from industrial sites be recruited for future study. Another limitation was that only EMG measurements were taken, and not real joint torque or joint load. In future studies, different approaches (e.g., force plates, motion study) should be applied to record joint moments and joint load. Recording postures and considering more muscles, such as the anterior deltoid, latissimus dorsi, and flexor digitorum, would allow more accurate estimation of overall muscular loadings and the construction of a mechanical model. Such information would be useful in understanding the mechanisms of various COMs in pushing and pulling a nursing cart. Finally, it must be noted that the tasks in the study were performed on a wooden floor of relatively constant friction, but in reality a nursing cart is generally used in medical care sites with quite a range of floor surfaces. In the future, different surface materials may be used to test the conditions of the actual work environment.

## Conclusion

In conclusion, the findings suggest that first, when operating a nursing cart, nursing staff should push the cart forward instead of pulling it backward; second, that nursing work environments should be designed without uphill paths wherever possible; and finally, that in a nursing cart, the load should be distributed such that heavier materials are near the handle and in the middle and bottom of the cabinet to reduce the physical load during the operation of a nursing cart. The findings of this study should be of use in designing a better nursing cart and enhancing the performance of related work operations.
